# The New Path to Improve Construction Safety Performance in China: An Evolutionary Game Theoretic Approach

**DOI:** 10.3390/ijerph16132443

**Published:** 2019-07-09

**Authors:** Zongjie Pi, Xin Gao, Linyan Chen, Jinghua Liu

**Affiliations:** School of Economics and Management, Tongji University, Shanghai 200092, China

**Keywords:** safety performance, construction industry, evolutionary game

## Abstract

Evidence shows that there are many work-related accidents and injuries happening in construction projects and governments have taken a series of administrative measures to reduce casualties in recent years. However, traditional approaches have reached a bottleneck due to ignoring market forces, and thus new measures should be conducted. This study develops a perspective of safety performance (SP) for construction projects in China and puts forward a conception of the safety information system by using several brainstorming sessions to strengthen the safety supervision of participants in the construction industry. This system provides rating information to the public, and bad performance contractors enter into a blacklist which will influence their economic activities. Considering the limited rationality of government and various contractors, this paper builds a reasonable evolutionary game model to verify the feasibility of the safety information system. The analysis results show that there is not a single set of evolutionarily stable strategies (ESSs), as different situations may lead to different ESSs. The efficiency of applying the safety information system (the blacklist) in the construction industry can be proved by reducing the government’s safety supervision cost and by enhancing construction safety at the same time.

## 1. Introduction

While the construction industry plays a significant role in promoting urbanization, it is also an accident-prone industry because of its complex site conditions, many risk factors, and high mortality rate [1,2]. Based on global statistics, the risk of a fatal accident in the construction industry is five times higher than that of other industries, and the casualty rate is three times higher [3,4,5]. For example, in the United States, although the construction industry accounts for less than 8% of the total labor force, work-related fatalities in construction industry make up over 22% of all occupational fatalities [6]. When compared with all industries, the construction industry takes up 27.6% and 31% of all work fatalities caused by occupational incidents in Korea and the United Kingdom, respectively [7,8]. The safety and health of workers in construction industry have become an important public health problem in recent decades. As life is the foundation of human existence, safety management should be the most important management function in the construction industry [9].

Therefore, many studies have been done to determine the main contributing factors in construction safety management, trying to explore effective methods to improve safety performance and reduce casualties. In recent decades, it has been well proven that safety climate was associated with safety behavior and occupational injuries in the construction site [10]. Moreover, it was found that safety climate was directly related to safety performance [11,12] and had an indirect influence on occupational injury through safety performance [13]. Measurements of safety climate and employees’ participation were found to help improve safety performance [12]. Once the employees were aware of the importance of safety issues and felt empowered to the project, they kept the construction project clean and orderly [14]. The safety rules and regulations were also significant for occupational incidents prevention and incident cost reduction [15]. Hong Kong emphasizes legislative changes, including the legal procedures related to site safety, and a range of systems and techniques that can be applied into construction safety management. The United States and the United Kingdom implemented a comprehensive safety supervision mode to strengthen communication between government departments and industry associations. Unlike other countries and regions, China relies more on administrative means to realize construction safety. There are three important laws that apply to construction safety: the Labor Law, the Construction Law and the Law of Working Safety in China [16]. The Administrative Regulations on the Work Safety of Construction Projects have more detailed rules in construction safety [17]. Although the government has introduced various safety rules and regulations, as well as spending a lot of effort to safety management in recent years, the accident rate in the construction industry still remains relatively high [15]. According to relevant statistics, there were 734 cases of housing and municipal engineering production safety accidents and 840 deaths in the year 2018, an increase of 42 from the number of accidents and an increase of 33 deaths during 2017 in China. That is because the central government implemented a single government supervision mode, which is based on territorial management and grading responsibility [18]. It is difficult for nongovernmental organizations (such as associations) to participate in the core work of safety supervision. The defects of this “nanny-style” regulation are as follows:

- On the one hand, the human and material resources which the government invests in safety are higher than the actual control effect. On the other hand, the efficiency of safety regulation is low because it relies solely on government forces and fails to optimize the allocation of social resources.
- It establishes the antagonistic relationship between the supervisors and the regulated parties, so it cannot effectively raise the enthusiasm and consciousness of participants’ responsibility, which is not conducive to the sustainable and healthy development of safety supervision.

This paper mainly focuses on two questions. The first question is how to find new path to improve construction safety performance in China. The second question is to explore how this new path affects the decision-making behavior of participants. Among the participants involved in the construction projects, contractors are considered as the most important management objects of the government and have direct responsibility for safety construction. This means that the contractors’ safety work is the key to safe construction, which can avoid the greatest safety risks and reduce the adverse impact of safety accidents. In order to facilitate the establishment of research models, we abstracted the practical problems in a rational way. Therefore, in this paper, the game participants are simplified into the government and contractors. Based on the defects of “nanny-style” regulation, it is an urgent task to further strengthen the management level of safety supervision by enhancing contractors’ sense of responsibility.

On this basis, this paper provides a new path to toughen the contractors’ responsibility for safety construction—to apply the safety information system in the construction industry. The safety information system will provide rating information to the public, which means contractors with bad performance will be added into a blacklist. Thus, contractors’ economic activities will be seriously influenced by the rating because every participant involved in construction projects can observe contractors’ safety credit records according to this information system. As the contractors make extensive use of the cost-benefit analysis when making safety effort decisions [19], the huge economic losses caused by blacklist records will stimulate them to pay more attention to their safety performance. Considering the limited rationality of government and various contractors, evolutionary game theory is conducted to verify the feasibility and effectiveness of this new path to improve construction safety performance in China. By exploring the evolutionarily stable strategies (ESSs) of government and contractors in the safety information system, the decision-making behaviors of participants are classified in 5 situations with 12 different cases, all of which are discussed in detail and are clearly identified from an evolutionary game perspective. This would allow us to understand the strategies of participants in specific situations clearly and identify key factors in the safety information system to promote safety performance.

This paper is organized as follows: the second section of this research paper is a literature review about safety performance and the blacklist. The third section proposes the concept of the safety information system, defines participants’ duties and clarifies the management process. The fourth section establishes an evolutionary game model, and a detailed analysis of the parameters is conducted in this part. The fifth section discusses two ultimate strategies and how the safety information system helps to achieve optimization of this model. In conclusion, some constructive implications for the government and limitations are given.

## 2. Literature Review

Safety performance can be defined as “actions or behaviors that individuals exhibit in almost all jobs to promote the health and safety of workers, clients, the public, and the environment” [20]. Many different programs, techniques, and initiatives can be implemented to enhance safety performance in the construction industry [11]. The path mentioned the most to improve safety performance in current research is to strengthen safety climate [14,21,22].

Safety climate refers to the perception of employees on safety-related organizational policies, procedures, and practices [21]. There is a consensus that workplace accidents will be reduced if contractors increase safety awareness, which means improving the safety climate is an effective route to better safety performance [20,21,23,24,25,26]. In the construction industry, a number of notable safety climate studies have been conducted [22,27,28,29,30]. Researchers have the perception that safety climate factors could be applied as a means of determining safety performance. The Nordic Safety Climate Questionnaire (NOSACQ-50) consisting of 50 items was used and seven dimensions were identified [31]: (1) management safety priority, commitment, and competence; (2) management safety empowerment; (3) management safety justice; (4) workers’ safety commitment; (5) workers’ safety priority and risk non-acceptance; (6) safety communication, learning, and trust in coworkers’ safety competence; and (7) workers’ trust in the efficacy of safety systems. In reference [2], three safety climate factors were identified: (1) management and employee commitment to occupational health and safety; (2) application of safety rules and work practices; and (3) responsibility for the health and safety. In reference [32], four factors were identified: (1) management commitment and employees’ involvement in health and safety; (2) safety enforcement and promotion; (3) applicability of safety rules and safe work practices; (4) safety consciousness and responsibility. The above three classifications of different safety climate factors reflected the importance of workers’ safety awareness. Therefore, workers’ safety behaviors were always at the core of improving the safety climate. Other studies evaluated safety performance through different techniques, including accident rates for work sites and injuries data [33,34], self-reported injury data collected through a questionnaire [35] and so on.

Other paths to improve safety performance include enhancing safety work, increasing safety investment and some administrative methods. A positive correlation was confirmed between safety work and safety performance, including indicators such as accident frequency and accident severity [36,37,38]. They also pointed out that the impact of safety work on safety performance was regulated by factors such as the degree of hazard and safety culture within the organization. Meanwhile, an inverse relationship was confirmed between the average number of accidents and the accident prevention cost [39]. Therefore, several basic models about safety efforts have been proposed to minimize the cost of accident prevention [25,40,41,42]. As one of the administrative methods available, taxation was considered a common method to improve safety performance. Governments have pushed enterprises to allocate resources equally between safety efforts and construction production by imposing a tax [43,44]. In reference [45], the result showed that safety regulations and rules were effective for safety performance to some extent. The Pay for Safety Scheme was one of the safety regulations and rules in Hong Kong which was an effective safety incentive. It was launched in the public sector by the Hong Kong government in 1996 [46,47].

However, research also shows that a plateau has been reached in safety improvement [35]. Existing research has not been strong enough to solve the defects of “nanny-style” regulation, as the majority of existing studies focused on how to strengthen government forces through safety regulations [48]. But there are few studies on improving safety performance through the forces of market mechanisms, on interactions between government and contractors or the competitive behaviors between different contractors. Therefore, in addition to the mandatory supervision mode of government, it is necessary to explore the supplementary supervision methods with the help of market forces. In reference [49], the Brazilian Ministry of Environment used a “district blacklist” as a measurable option for reducing Amazon forest loss, and the blacklist has considerably reduced deforestation in the affected districts. The blacklisting strategy is also used in the field of Safe Browsing, where the main defense against phishing and malware attacks is based on blacklists [50]. According to a recent study, the blacklisting strategy can be a great help in engaging project participants in governance [51]. Therefore, the Chinese government has the intention to implement the Bad Record System of Safety Production (the blacklist system) among construction enterprises as a part of a social credit system to improve safety performance.

Based on previous research, the significance of this study lies in introducing the blacklist as a new path for improving construction safety performance in China with an evolutionary game theoretic approach. The blacklist system not only serves as a governance mechanism based on the market but also an effective supplement to government regulation which could be verified by the evolutionary game model that mainly contributes to the following aspects:

(1) It enlarges the current regulatory effectiveness of the government, embodying the multiplicity and long-term effectiveness of the supervision effect.
(2) It forces contractors to accomplish their safety responsibilities and enhances the safety awareness of self-management of all participating units.
(3) All parties in the society participate in co-management and form a good safe production environment.

## 3. The Conception of Safety Information System

A construction safety information system has been developed for general safety managers and workers [52]. For all the contractors in the construction industry, it would be efficient to build a safety information system to regulate them.

Brainstorming is an effective method to create innovative ideas using the wisdom of groups. Besides our research team, the brainstorming participants comprised professionals from different institutions. These institutions could be divided into three categories: the government, the association, and the contractors. We have organized many seminars and fully discussed the conception of a safety information system, which greatly promoted the research process. In the first seminar, professionals from different institutions first proposed the concept of establishing a safe record system for safety production of construction enterprises, namely a safety information system. In the following seminars, we discussed and confirmed the application scope of the safety information system, as well as the basic principles, the duties of participants, and the standard information collection process. In the final stage, we asked for opinions and suggestions from the construction industry practitioners in three typical cities in China. Our research team investigated the safety assessment method of the local safety supervision department and the establishment process of the information system, in order to draw on the successful experience of the construction of the safe record system and strive to promote the safety information system nationwide.

### 3.1. Construction Participants and Their Duties

**Figure 1:**
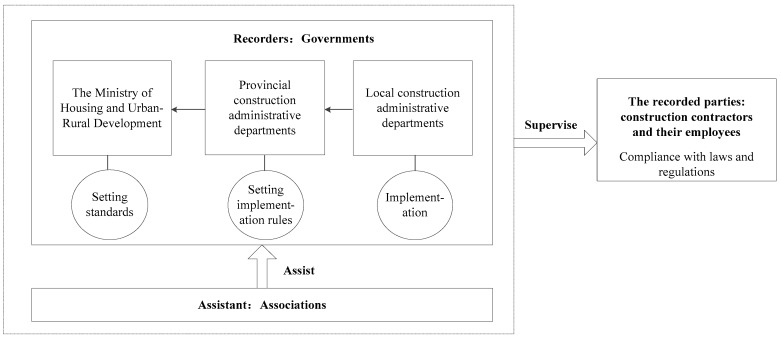
Relationships among participants and their duties.

The main construction participants in the safety information system are three parties: governments, associations, and construction contractors. Governments are more authoritative, and their participation is more necessary than other participants. Therefore, we have defined the role of governments as recorders, aggregators and publishers. Associations assist governments to discipline the industry by improving the industry’s credit self-discipline standards and improving the industry’s integrity mechanism. As the recorded parties, construction contractors and their employees should comply with laws and regulations. Three participants interact and restrict with one another. Their relationship is shown in Figure 1.

The governments are divided into three levels: the Ministry of Housing and Urban-Rural Development, the provincial construction administrative departments and the local construction administrative departments. The local construction administrative departments record the behaviors of construction contractors who break their promise and report to provincial construction administrative departments. Besides setting implementation rules, the provincial construction administrative departments collect information and report to the Ministry of Housing and Urban-Rural Development. The highest level would disclose the information to the public.

### 3.2. Management Process

**Figure 2:**
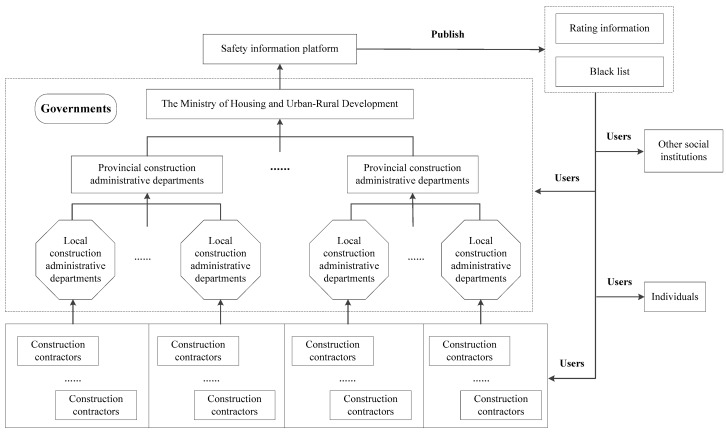
Structure of safety information system in construction industry.

Complete, timely and reliable safety information is the foundation of the safety information system. The safety information collected by governments from construction contractors is uploaded to the safety information system and updated in real time. According to the information, the safety information system will automatically rate the contractors. Some contractors who have bad performance in safety issues will be put on the blacklist, which means that their business will be affected. The structure of the safety information system in the construction industry is shown in Figure 2.

The users of the safety information system in the construction industry will be divided into four parts: governments, construction contractors, other social institutions and individuals. Governments could manage contractors in a targeted way, such as strengthening the supervision of blacklisted contractors. The contractors are also users of the safety information system because subcontracting is common in this industry. If the subcontractors have serious incidents during the construction project, contractors also need to take responsibility for it. This is the reason why safety information is significant for contractors. Other social institutions which will have cooperation with contractors have access to the safety information in advance to make the decision about whether to cooperate or not. When looking for a job, the individuals could search for safety information about target contractors so as to enjoy a better working environment.

## 4. Evolutionary Game Model between Government and Contractors

Evolutionary games were first introduced to describe the evolution of nature lives by biologists [53]. In traditional game theory, the game players are supposed to be rational and they are interdependent with other fully rational players [54,55,56]. However, it is almost impossible for players to maintain rationality in every game process [53]. Players in evolutionary games are supposed to learn how to play through experience. These players have bounded rationality and are not the perfect rational persons that are usually assumed to exist in traditional game theory [57,58,59]. In addition, evolutionary games still set up the payoff matrix objectively and analyze it through the classical expected utility theory [54].

When the safety information system is put into practice, the government and contractors start to influence each other’s decisions. The interaction between them could be regarded as a dynamic game process, because players in an evolutionary game engage in multiple rounds of interaction by adopting different strategies and their interaction state varies from replication games. Therefore, the evolutionary games provide a rational and effective way to introduce the replicator dynamics mechanism where optimal strategies are copied by others and spread in interactive rounds [60,61]. In this paper, we set up an evolutionary game model between the government and contractors which can be used to analyze the optimal strategies of players.

### 4.1. Model Assumptions

Before constructing the model, it is vital for us to consider the relationship between the reality of safety information system in the operational period and the hypothetical model. Therefore, we must provide the following assumptions to ensure the objectivity and scientific nature of the evolutionary game model:

**Assumption** **1.** *There are only two players in the game: the government and contractors. Both of them have independent decision-making ability and a mastery of their information level. As a result, players have limited rationality and choose behavioral strategies independently based on the value of their own strategies. Over the whole game process, they can change their decisions dynamically.*

**Assumption** **2.** *The contractor has two behavioral strategies: the first is to obey the rules; the other is not to, particularly not to follow the relevant contractual and policy regulations. This behavioral strategy will obtain certain additional benefits, but it will cause certain detriments to their social interests at the same time. Therefore, the behavioral strategies for contractor are {obey, not obey}. The government also has two strategies: one is to conscientiously supervise the behavior of contractors; the other is not to supervise. The supervision strategy can restrain the contractor’s irregularities to a certain extent, and reduce the contractor’s additional income, but it will also generate certain supervision costs. Therefore, the behavioral strategies for government are {supervise, not supervise}.*

**Assumption** **3.** *There is no collusion existing during this game period. For instance, if the government chooses to supervise, contractors would be caught immediately as long as they violate the rules, that is to say, the success rate of the government in detecting the contractor’s irregularities during the game period is one hundred percent.*

**Assumption** **4.** *The government values social benefits generated by the project, while contractors pursue economic benefits. Over the whole game process, players choose their own behavioral strategies in terms of how much interest they can obtain, regardless of the interest changes caused by other external factors in the environment.*

### 4.2. Payoff Matrix and Parameters

The government aims to maximize the overall social benefits. There are two behavioral strategies the government can take in the safety supervision: one is to supervise the contractors regularly according to the rules, the other is not to supervise the contractors. Therefore, the set of behavioral strategies which the government can choose from is {supervise, not supervise}. Meanwhile, the contractors will prioritize the strategy that can maximize their revenue and they also have two behavioral strategies: one is to obey the rules. It means contractors would discipline themselves to construct safely. The other is not to obey the rules. Contractors are opportunists who are busy pursuing the greatest benefits attainable with the perception that safety construction costs are too high to ensure their profits. Therefore, the set of behavioral strategies that contractors can choose from is {obey, not obey}. Relevant parameters explained as follows in Table 1 are non-negative numbers in the payoff matrix.

If they obey the rules, a contractor’s revenue is $\left(R-\alpha_{1}A\right)$, and they pay Ce for safety construction. Thus, their net income is $\left(R-Ce-\alpha_{1}A\right)$. In a sense, government revenue mainly refers to taxes that are closely related to the normal business revenue of contractors. If the government do not supervise contractors, the net income of the government is $\left(k_{1}R-k_{2}^{\prime }\alpha_{1}A\right)$. If the government supervises contractors, there will be an extra cost Cg for safety supervision, which is mainly spending on the inspection of the construction site. Thus, the net income of the government is $\left(k_{1}R-Cg-k_{2}\alpha_{1}A\right)$.

If contractors do not obey the rules and the government does not supervise, contractors only consider the loss caused by safety accidents, so their net income is easy to figure out, it is $\left(R-\alpha_{2}A\right)$. At the same time, if the government does not supervise contractors, the net income of the government is $\left(k_{1}R-k_{2}^{\prime }\alpha_{2}A\right)$. But if contractors do not obey the rules under the government’s supervision, contractors would be listed on the blacklist and pay for a series of administrative penalties, which will damage the reputation of these contractors in the market. Thus, their normal business revenue would decrease. In this situation, the net income of contractors is $\left(R^{\prime }-\alpha_{2}A-P_{1}\right)$, and the government would have an additional income, which is the fine paid by contractors.

Considering the relationship between government and contractors, a payoff matrix is shown in Table 2. The first entry of a cell is the payoff for the contractors, while the second entry is the payoff for the government.

### 4.3. Model Establishment

We suppose that the proportion of contractors choosing to pay attention to safety issues (i.e., obey the rules) is x(0<x<1), thus the proportion of contractors choosing not to pay attention to safety issues is 1−x. Similarly, the probability of government supervising contractors is y(0<y<1), and the probability of government not supervising contractors is 1−y.

It is assumed that the expected gain when contractors obey the rules is $U_{11}$, and the expected gain when contractors do not obey the rules is $U_{12}$. The mean expected gain for the entire contractor group is $U_{1}$. The representative equations, respectively, are as follows:

(1) $$U_{11}=(R-Ce-\alpha_{1}A)y+(R-Ce-\alpha_{1}A)(1-y)=R-Ce-\alpha_{1}A$$

(2) $$U_{12}=(R^{\prime }-\alpha_{2}A-P_{1})y+(R-\alpha_{2}A)(1-y)=-(R-R^{\prime }+P_{1})y+R-\alpha_{2}A$$

(3) $$U_{1}=xU_{11}+(1-x)U_{12}=[(R-R^{\prime }+P_{1})y+(\alpha_{2}-\alpha_{1})A-Ce]x-(R-R^{\prime }+P_{1})y+R-\alpha_{2}A$$

Similarly, it is assumed that the expected gain when the government adopts a supervision strategy is $U_{21}$, and the expected gain when the government chooses a strategy not to supervise is $U_{22}$. The mean expected gain for the entire government group is $U_{2}$. The representative equations, respectively, are as follows:

(4) $$\begin{array}{ll} U_{21} & =(k_{1}R-Cg-k_{2}\alpha_{1}A)x+(k_{1}R^{\prime }-Cg-k_{2}\alpha_{2}A+P_{2})(1-x)\\ & =[k_{1}(R-R^{\prime })+(\alpha_{2}-\alpha_{1})k_{2}A-P_{2}]x+(k_{1}R^{\prime }-Cg-k_{2}\alpha_{2}A+P_{2}) \end{array}$$

(5) $$\begin{array}{ll} U_{22} & =(k_{1}R-k_{2}^{\prime }\alpha_{1}A)x+(k_{1}R-k_{2}^{\prime }\alpha_{2}A)(1-x)\\ & =(\alpha_{2}-\alpha_{1})k_{2}^{\prime }Ax+(k_{1}R-k_{2}^{\prime }\alpha_{2}A) \end{array}$$

(6) $$\begin{array}{ll} U_{2} & =yU_{21}+(1-y)U_{22}\\ & =\left\{[k_{1}(R-R^{\prime })-(k_{2}^{\prime }-k_{2})(\alpha_{2}-\alpha_{1})A-P_{2}]x-k_{1}(R-R^{\prime })+(k_{2}^{\prime }-k_{2})\alpha_{2}A-Cg+P_{2}\right\}y+\\ & (\alpha_{2}-\alpha_{1})k_{2}^{\prime }Ax+k_{1}R-k_{2}^{\prime }\alpha_{2}A \end{array}$$

### 4.4. Model Solution

The core of an evolutionary game is the dynamic change of strategy proportion. The change rate is significant, because the sign represents the changing direction of the proportion. A positive sign means the effectiveness of the strategy is increasing and a negative sign has the opposite meaning. We can determine a stable state of this model and possible equilibrium points in this method.

According to Equations (1)–(3), the replicator dynamics equation that determines the proportion of contractors who obey the rules could be represented as follows. The parameter t means the time, and dx/dt is the change rate of the proportion of contractors who obey the rules over time.

(7) $$F(x)=\frac{dx}{dt}{=x(U}_{11}-{\bar{U}}_{1})=x(1-x)[(R-R^{\prime }+P_{1})y+(\alpha_{2}-\alpha_{1})A-Ce]$$

Similarly, according to Equations (4)–(6), the replicator dynamics equation that determines the probability of government that supervises contractors could be represented as follows. dy/dt is the change rate of the probability of government which supervises contractors.

(8) $$\begin{array}{ll} F(y) & =\frac{dy}{dt}{=y(U}_{21}-{\bar{U}}_{2})\\ & \;=y(1-y)\left\{[k_{1}(R-R^{\prime })-{(k}_{2}^{\prime }-k_{2})(\alpha_{2}-\alpha_{1})A-P_{2}]x-k_{1}(R-R^{\prime })+(k_{2}^{\prime }-k_{2})\alpha_{2}A-Cg+P_{2}\right\} \end{array}$$

Friedman (1991) proposed a method to derive stability conditions at an equilibrium point through the Jacobi matrix. The judging standard of ESS is shown in Table 3.

(9) $$J=\left(\begin{array}{cc} \frac{\partial F(x)}{\partial x} & \frac{\partial F(x)}{\partial y}\\ \frac{\partial F(y)}{\partial x} & \frac{\partial F(y)}{\partial y} \end{array}\right)$$

When the dynamic equations equal 0, the equations will no longer evolve, and the system has reached an equilibrium point. We could derive five possible equilibrium points of (x,y): $E_{1}\left(0,0\right)$, $E_{2}\left(0,1\right)$, $E_{3}\left(1,0\right)$, $E_{4}\left(1,1\right)$, $E_{5}\left(x*,y*\right)$, where x*= $\frac{\left(k_{2}^{\prime }-k_{2}\right)\alpha_{2}A-k_{1}(R-R^{\prime })-Cg+P_{2}}{\left(k_{2}^{\prime }-k_{2}\right)\left(\alpha_{2}-\alpha_{1}\right)A-k_{1}(R-R^{\prime })+P_{2}}$ and y*= $\frac{Ce-\left(\alpha_{2}-\alpha_{1}\right)A}{R-R^{\prime }+P_{1}}$.

Its determinant equation and its trace of this Jacobi matrix could be derived. Bring the five possible equilibrium points into the determinant equation and the trace of the Jacobi matrix. The result has been shown in Table 4.

After observation, the signs of det*J* and tr*J* are determined by four parts. A stable point of this system is also determined by the following four parts: $[\left(\alpha_{2}-\alpha_{1}\right)A-Ce]$, $\left[R-R^{\prime }+P_{1}+\left(\alpha_{2}-\alpha_{1}\right)A-Ce\right]$, $\left[(k_{2}^{\prime }-k_{2})\alpha_{2}A-k_{1}(R-R^{\prime })-Cg+P_{2}\right]$ and $\left[(k_{2}^{\prime }-k_{2})\alpha_{1}A-Cg\right]$. To improve the readability of this article, we use a, b, c, d to represent these formulas. A further explanation can be seen in Table 5. Because the sign of $\left(R-R^{\prime }\right)$ is positive and a is definitely smaller than b. However, the size relationship between c and d is not clear.

**Figure 3:**
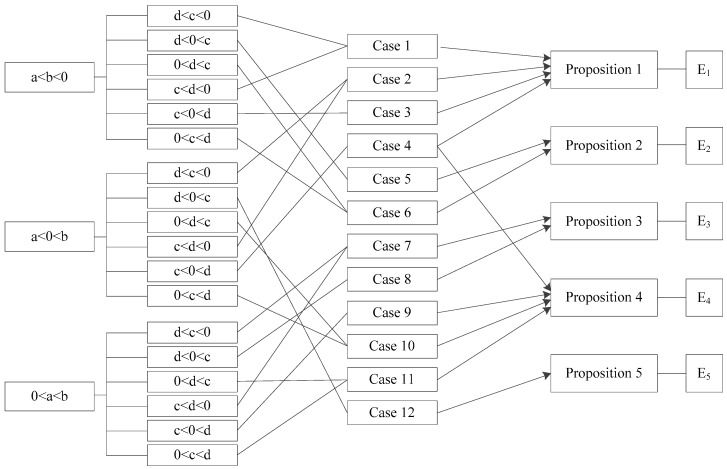
Diagram of classification.

From the discussion above, we could not determine the signs of these formulas easily. Therefore, we conducted a classification discussion based on the size relationship of a, b, c and d. The specific classification process is divided into the following three stages. On the first stage, under the premise that a is definitely smaller than b, we classify the size of these four symbols into 18 basic circumstances. We further derive the evolution path of each circumstance and its evolutionary stable equilibrium point. On the second stage, in order to simplify and clarify the discussion process, we combined some of these circumstances in line with the conditions when a stable equilibrium state is reached. Therefore, the 18 basic circumstances are classified into 12 cases. We could derive 12 formula combinations to conduct further analysis into this question, and then comes to the third stage. At this stage, we classify these 12 cases according to five possible equilibrium points: $E_{1}\left(0,0\right)$, $E_{2}\left(0,1\right)$, $E_{3}\left(1,0\right)$, $E_{4}\left(1,1\right)$, $E_{5}\left(x*,y*\right)$, and finally get five corresponding propositions. The specific classification process is shown in Figure 3.

### 4.5. Model Analysis

As it is mentioned, whether the possible equilibrium point is stable depends on the signs of det*J* and tr*J*. We conduct an analysis of the local stability of equilibrium in twelve cases and try to find out the stable point of the system based on the judging standard. The diagrams on the dynamic evolution of the equilibrium points are given for further study.

**Proposition** **1.** *When a<0 and c<0, $E_{1}\left(0,0\right)$ is an ESS, which means the government and contractors will choose to {not supervise, not obey}.*

**Proof.**  This ESS can be subdivided into the following four cases (cases 1–4). □

Case 1: When a<b<0, c<0 and d<0.

On the one hand, when d<c<0, we could derive that the sign of (c−d) is positive and $x*=\frac{c}{c-d}<0$, which is in contradiction with the probability meaning of x*. On the other hand, when c<d<0, we could derive that the sign of (b−a) is positive and $y*=\frac{-a}{b-a}>0$. While −a−(b−a)=−b>0, the value of y* is greater than 1 which is in contradiction with the probability meaning of y*.

**Figure 4:**
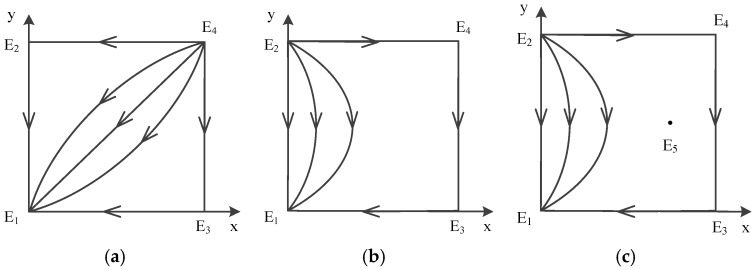

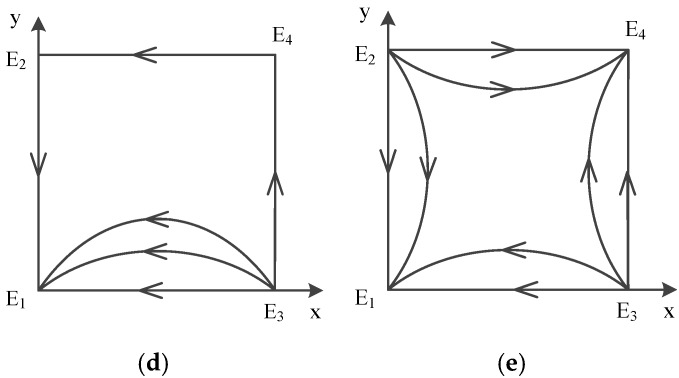
Diagram on dynamic evolution of equilibrium points in $E_{1}\left(0,0\right)$, (**a**) Case 1 (**b**) Case 2-1 (**c**) Case 2-2 (**d**) Case 3 (**e**) Case 4.

In a word, point $E_{5}\left(x*,y*\right)$ does not exist, and there are only four possible equilibrium points left: $E_{1}\left(0,0\right)$, $E_{2}\left(0,1\right)$, $E_{3}\left(1,0\right)$, $E_{4}\left(1,1\right)$. The analysis of local stability of equilibrium about these four points is shown in Table 6 (Case 1). According to the conclusion, the trace of dynamic evolution is depicted in Figure 4a, which shows dynamic evolution diagram of strategies between government and contractors.

We can see that the evolutionary model will eventually converge at $E_{1}\left(0,0\right)$ no matter which strategies are initially used by game players. Therefore, $E_{1}\left(0,0\right)$ is the evolutionarily stable point; $E_{2}\left(0,1\right)$ and $E_{3}\left(1,0\right)$ are saddle points; and $E_{4}\left(1,1\right)$ is the unstable point. The ESS is {not supervise, not obey}.

Case 2: When a<0<b, c<0 and d<0.

Same as the discussion above, when d<c<0, the sign of c−d is positive and the sign of, we could derive $x*=\frac{c}{c-d}<0$. Point $E_{5}\left(x*,y*\right)$ does not exist, there are only four possible equilibrium points left: $E_{1}\left(0,0\right)$, $E_{2}\left(0,1\right)$, $E_{3}\left(1,0\right)$, $E_{4}\left(1,1\right)$.

However, when c<d<0, the sign of (c−d) and c is negative, we could derive $x*=\frac{c}{c-d}>0$, otherwise c−(c−d)=d<0, thus $x*=\frac{c}{c-d}<1$. The value of x* belongs to [0, 1]. In addition, a<0 and b−a>0, we could derive $y*=\frac{-a}{b-a}>0$. −a−(b−a)=−b<0, we could derive $y*=\frac{-a}{b-a}<1$. The value of y* belongs to [0, 1].

Afterwards, point $E_{5}\left(x*,y*\right)$ exists, and there are five possible equilibrium points left: $E_{1}\left(0,0\right)$,$E_{2}\left(0,1\right)$, $E_{3}\left(1,0\right)$, $E_{4}\left(1,1\right)$, $E_{5}\left(x*,y*\right)$. The analysis of local stability of equilibrium about these four points is shown in Table 6 (Case 2-1), and about another five points are shown in Table 6 (Case 2-2). According to the conclusion, the evolution of mixed strategy is shown in Figure 4b,c, which depicts dynamic evolution diagram of strategies between government and contractors. The difference between case 1 and case 2 is the unstable point. The difference between the two circumstances in case 2 is the $E_{5}$ point.

We can see that the evolutionary model will eventually converge at $E_{1}\left(0,0\right)$ no matter which strategies are initially used by game players. Therefore, $E_{1}\left(0,0\right)$ is the evolutionarily stable point; $E_{2}\left(0,1\right)$ and $E_{3}\left(1,0\right)$ are saddle points; and $E_{4}\left(1,1\right)$ is the unstable point; in case 2-2 $E_{5}\left(x*,y*\right)$ is the center point. The ESS is {not supervise, not obey}.

Case 3: When a<b<0 and c<0<d.

In this case, we could derive that the sign of b−a is positive and $y*=\frac{-a}{b-a}>0$. While −a−(b−a)=−b>0, the value of y* is greater than 1 which is in contradiction with the probability meaning of y*.

In a word, point $E_{5}\left(x*,y*\right)$ does not exist. There are only four possible equilibrium points left: $E_{1}\left(0,0\right)$, $E_{2}\left(0,1\right)$, $E_{3}\left(1,0\right)$, $E_{4}\left(1,1\right)$. The analysis of local stability of equilibrium about these four points is shown in Table 6 (Case 3). According to the conclusion, the evolution of mixed strategy is shown in Figure 4d, which depicts dynamic evolution diagram of strategies between government and contractors.

We can see that the evolutionary model will eventually converge at $E_{1}\left(0,0\right)$ no matter which strategies are initially used by game players. Therefore, $E_{1}\left(0,0\right)$ is the evolutionarily stable point; $E_{2}\left(0,1\right)$ and $E_{4}\left(1,1\right)$ are saddle points; and $E_{3}\left(1,0\right)$ is the unstable point. The ESS is {not supervise, not obey}.

Case 4: When a<0<b and c<0<d.

In this case, c<0 and c−d<0, we could derive $x*=\frac{c}{c-d}>0$. Otherwise c−(c−d)=d>0, the value of x* is greater than 1 which is in contradiction with the probability meaning of x*.

In a word, point $E_{5}\left(x*,y*\right)$ does not exist, and there are only four possible equilibrium points left: $E_{1}\left(0,0\right)$, $E_{2}\left(0,1\right)$, $E_{3}\left(1,0\right)$, $E_{4}\left(1,1\right)$. The analysis of local stability of equilibrium about these four points is shown in Table 6 (Case 4). According to the conclusion, the evolution of mixed strategy is shown in Figure 4e, which depicts dynamic evolution diagram of strategies between government and contractors.

We can see that the evolutionary model will eventually converge at $E_{1}\left(0,0\right)$ and $E_{4}\left(1,1\right)$ no matter which strategies are initially used by game players. Therefore, $E_{1}\left(0,0\right)$ and $E_{4}\left(1,1\right)$ are the evolutionarily stable points; $E_{2}\left(0,1\right)$ and $E_{3}\left(1,0\right)$ are the unstable points. The ESSs are {not supervise, not obey} and {supervise, obey}.

According to the conclusion, the evolution of mixed strategy is shown in Figure 3, which depicts a dynamic evolution diagram of strategies between the government and contractors. It is worth mentioning the situation that both $E_{1}\left(0,0\right)$ and $E_{4}\left(1,1\right)$ are ESS points in case 4.

**Proposition** **2.** *When a<b<0 and c>0, $E_{2}\left(0,1\right)$ is an ESS, which means the government and contractors will choose to {not supervise, obey}.*

**Proof.**  This ESS can be subdivided into the following two cases (case 5, case 6). □

Case 5: When a<b<0 and d<0<c.

In this case, we could derive $y*=\frac{-a}{b-a}>0$. While −a−(b−a)=−b>0, the value of y* is greater than 1 which is in contradiction with the probability meaning of y*.

**Figure 5:**
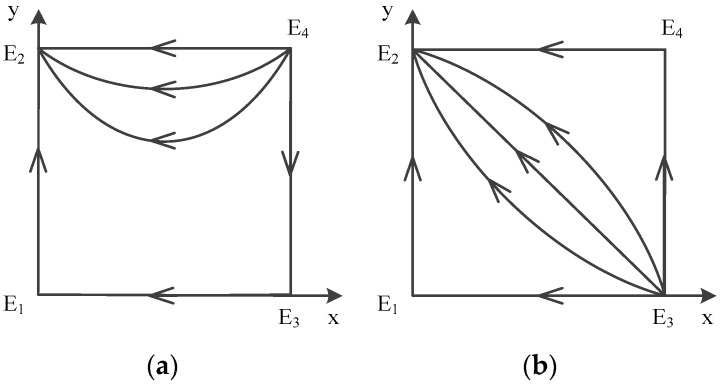
Diagram on dynamic evolution of equilibrium points in $E_{2}\left(0,1\right)$, (**a**) Case 5 (**b**) Case 6.

In a word, point $E_{5}\left(x*,y*\right)$ does not exist, and there are only four possible equilibrium points left: $E_{1}\left(0,0\right)$, $E_{2}\left(0,1\right)$, $E_{3}\left(1,0\right)$, $E_{4}\left(1,1\right)$. The analysis of local stability of equilibrium about these four points is shown in Table 7 (Case 5). According to the conclusion, the evolution of mixed strategy is shown in Figure 5a, which depicts dynamic evolution diagram of strategies between government and contractors.

We can see that the evolutionary model will eventually converge at $E_{2}\left(0,1\right)$ no matter which strategies are initially used by game players. Therefore, $E_{2}\left(0,1\right)$ is the evolutionarily stable point; $E_{1}\left(0,0\right)$ and $E_{3}\left(1,0\right)$ are saddle points; and $E_{4}\left(1,1\right)$ is the unstable point. The ESS is {not supervise, obey}.

Case 6: When a<b<0, c>0 and d>0.

On the one hand, when 0<d<c, we could derive c−d>0 and d>0, thus $x*=\frac{c}{c-d}>0$. Otherwise c−(c−d)=d>0, the value of x* is greater than 1 which is in contradiction with the probability meaning of x*. On the other hand, when 0<c<d. We could derive c>0 and c−d<0, thus $x*=\frac{c}{c-d}<0$, which is in contradiction with the probability meaning of x*.

In a word, point $E_{5}\left(x*,y*\right)$ does not exist, and there are only four possible equilibrium points left: $E_{1}\left(0,0\right)$, $E_{2}\left(0,1\right)$, $E_{3}\left(1,0\right)$, $E_{4}\left(1,1\right)$. The analysis of local stability of equilibrium about these four points is shown in Table 7 (Case 6). According to the conclusion, the evolution of mixed strategy is shown in Figure 5b, which depicts a dynamic evolution diagram of strategies between government and contractors.

We can see that the evolutionary model will eventually converge at $E_{2}\left(0,1\right)$ no matter which strategies are initially used by game players. Therefore, $E_{2}\left(0,1\right)$ is the evolutionarily stable point; $E_{1}\left(0,0\right)$ and $E_{4}\left(1,1\right)$ are saddle points; and $E_{3}\left(1,0\right)$ is the unstable point. The ESS is {not supervise, obey}.

According to the conclusion, the evolution of mixed strategy is shown in Figure 4, which depicts dynamic evolution diagram of strategies between government and contractors.

**Proposition** **3.** *When 0<a<b and d<0, $E_{3}\left(1,0\right)$ is an ESS, which means the government and contractors will choose to {supervise, not obey}.*

**Proof.**   This ESS can be subdivided into the following two cases (case 7, case 8). □

Case 7: When 0<a<b, d<0 and c<0.

On the one hand, when d<c<0. The sign of (c−d) is positive and the sign of c is negative, we could derive $x*=\frac{c}{c-d}<0$. On the other hand, when c<d<0. We could derive that the sign of (b−a) is positive and $y*=\frac{-a}{b-a}<0$.

**Figure 6:**
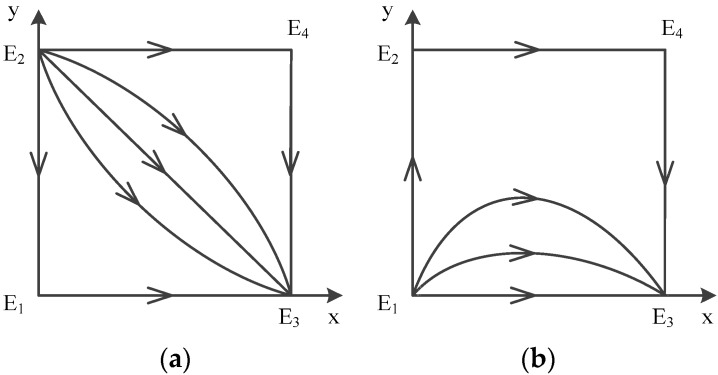
Diagram on Dynamic Evolution of Equilibrium Points in $E_{3}\left(1,0\right)$, (**a**) Case7 (**b**) Case 8.

In a word, point $E_{5}\left(x*,y*\right)$ does not exist, and there are only four possible equilibrium points left:$E_{1}\left(0,0\right)$, $E_{2}\left(0,1\right)$, $E_{3}\left(1,0\right)$, $E_{4}\left(1,1\right)$. The analysis of local stability of equilibrium about these points is shown in Table 8 (Case 7). According to the conclusion, the evolution of mixed strategy is shown in Figure 6a, which depicts a dynamic evolution diagram of strategies between government and contractors.

We can see that the evolutionary model will eventually converge at $E_{3}\left(1,0\right)$ no matter whichever strategies are initially used by game players. Therefore, $E_{3}\left(1,0\right)$ is the evolutionarily stable point; $E_{1}\left(0,0\right)$ and $E_{4}\left(1,1\right)$ are saddle points; and $E_{2}\left(0,1\right)$ is the unstable point. The ESS is {supervise, not obey}.

Case 8: When 0<a<b and d<0<c.

In this case, we could derive that the sign of (b−a) is positive and $y*=\frac{-a}{b-a}<0$, which is in contradiction with the probability meaning of y*.

In a word, point $E_{5}\left(x*,y*\right)$ does not exist, and there are only four possible equilibrium points left: $E_{1}\left(0,0\right)$, $E_{2}\left(0,1\right)$, $E_{3}\left(1,0\right)$, $E_{4}\left(1,1\right)$. The analysis of local stability of equilibrium about these four points is shown in Table 8 (Case 8). According to the conclusion, the evolution of mixed strategy is shown in Figure 6b, which depicts a dynamic evolution diagram of strategies between government and contractors.

We can see that the evolutionary model will eventually converge at $E_{3}\left(1,0\right)$ no matter what strategies are initially used by game players. Therefore, $E_{3}\left(1,0\right)$ is the evolutionarily stable point; $E_{2}\left(0,1\right)$ and $E_{4}\left(1,1\right)$ are saddle points; and $E_{1}\left(0,0\right)$ is the unstable point. The ESS is {supervise, not obey}.

According to the conclusion, the evolution of mixed strategy is shown in Figure 5, which depicts dynamic evolution diagram of strategies between government and contractors.

**Proposition** **4.** *When b>0 and d>0, $E_{4}\left(1,1\right)$ is an ESS, which means the government and contractors will choose to {supervise, obey}.*

**Proof.**  This ESS can be subdivided into the following four cases (case 4, case 9-case 11). □

Case 4: According to previous calculations, $E_{4}\left(1,1\right)$ is an ESS in case 4.

Case 9: When 0<a<b and c<0<d.

In this case, c−d<0 and c<0, we could derive $x*=\frac{c}{c-d}>0$. Because c−(c−d)=d>0, the value of x* is greater than 1 which is in contradiction with the probability meaning of x*.

**Figure 7:**
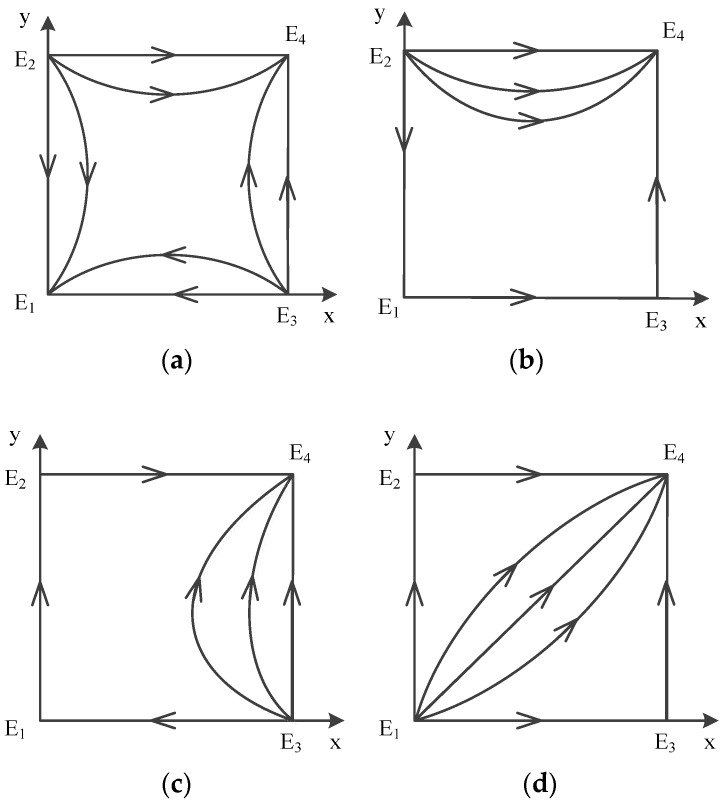
Diagram on Dynamic Evolution of Equilibrium Points in $E_{1}\left(0,0\right)$, (**a**) Case 4 (**b**) Case 9 (**c**) Case 10 (**d**) Case 11.

In a word, point $E_{5}\left(x*,y*\right)$ does not exist, and there are only four possible equilibrium points left: $E_{1}\left(0,0\right)$, $E_{2}\left(0,1\right)$, $E_{3}\left(1,0\right)$, $E_{4}\left(1,1\right)$. The analysis of local stability of equilibrium about these four points is shown in Table 9 (Case 9). According to the conclusion, the evolution of mixed strategy is shown in Figure 7b, which depicts a dynamic evolution diagram of strategies between the government and contractors.

We can see that the evolutionary model will eventually converge at $E_{4}\left(1,1\right)$ no matter whichever strategies are initially used by game players. Therefore, $E_{4}\left(1,1\right)$ is the evolutionarily stable point; $E_{1}\left(0,0\right)$ and $E_{3}\left(1,0\right)$ are saddle points; and $E_{2}\left(0,1\right)$ is the unstable point. The ESS is {supervise, obey}.

Case 10: When a<0<b, c>0 and d>0.

On the one hand, when 0<d<c, c>0 and c−d>0. We could derive $x*=\frac{c}{c-d}>0$. Otherwise c−(c−d)=d>0, the value of x* is greater than 1 which is in contradiction with the probability meaning of x*. On the other hand, when 0<c<d. Thus c>0 and c−d<0, we could derive $x*=\frac{c}{c-d}<0$.

In a word, point $E_{5}\left(x*,y*\right)$ does not exist, there are only four possible equilibrium points left: $E_{1}\left(0,0\right)$, $E_{2}\left(0,1\right)$, $E_{3}\left(1,0\right)$, $E_{4}\left(1,1\right)$. The analysis of local stability of equilibrium about these four points is shown in Table 9 (Case 10). According to the conclusion, the evolution of mixed strategy is shown in Figure 7c, which depicts a dynamic evolution diagram of strategies between the government and contractors.

We can see that the evolutionary model will eventually converge at $E_{4}\left(1,1\right)$ no matter whichever strategies are initially used by game players. Therefore, $E_{4}\left(1,1\right)$ is the evolutionarily stable point; $E_{1}\left(0,0\right)$ and $E_{2}\left(0,1\right)$ are saddle points; and $E_{3}\left(1,0\right)$ is the unstable point. The ESS is {supervise, obey}.

Case 11: When 0<a<b, c>0 and d>0.

On the one hand, when 0<d<c. Thus c>0 and c−d>0, we could derive $x*=\frac{c}{c-d}>0$. Otherwise c−(c−d)=d>0, the value of x* is greater than 1 which is in contradiction with the probability meaning of x*. On the other hand, when 0<c<d, c>0 and c−d<0, we could derive $x*=\frac{c}{c-d}<0$.

In a word, point $E_{5}\left(x*,y*\right)$ does not exist, and there are only four possible equilibrium points left: $E_{1}\left(0,0\right)$, $E_{2}\left(0,1\right)$, $E_{3}\left(1,0\right)$, $E_{4}\left(1,1\right)$. The analysis of local stability of equilibrium about these four points is shown in Table 9 (Case 11). According to the conclusion, the evolution of mixed strategy is shown in Figure 7d, which depicts a dynamic evolution diagram of strategies between the government and contractors.

We can see that the evolutionary model will eventually converge at $E_{4}\left(1,1\right)$ no matter what strategies are initially used by game players. Therefore, $E_{4}\left(1,1\right)$ is the evolutionarily stable point; $E_{2}\left(0,1\right)$ and $E_{3}\left(1,0\right)$ are saddle points; and $E_{1}\left(0,0\right)$ is the unstable point. The ESS is {supervise, obey}.

According to the conclusion, the evolution of mixed strategy is shown in Figure 6, which depicts a dynamic evolution diagram of strategies between the government and contractors.

**Proposition** **5.** When a<0<b and d<0<c, $E_{5}\left(x*,y*\right)$ is an ESS which means the government and contractors will reach a mixed equilibrium point.

**Proof.**  There is only one case for this ESS (case 12). □

Case 12: In this case, c>0 and c−d>0. Thus $x*=\frac{c}{c-d}>0$. Otherwise c−(c−d)=d<0, we could derive $x*=\frac{c}{c-d}<1$. The value of x* belongs to [0, 1]. $\left[\left(\alpha_{2}-\alpha_{1}\right)A-Ce\right]<0$ and b−a>0, we could derive $y*=\frac{-a}{b-a}>0$.−a−(b−a)=−b<0, we could derive $y*=\frac{-a}{b-a}<1$. The value of y* belongs to [0, 1].

So there are five possible equilibrium points in this case which is special comparing other cases: $E_{1}\left(0,0\right)$, $E_{2}\left(0,1\right)$, $E_{3}\left(1,0\right)$, $E_{4}\left(1,1\right)$, $E_{5}\left(x*,y*\right)$. The analysis of local stability of equilibrium about these five points is shown in Table 10.

**Figure 8:**
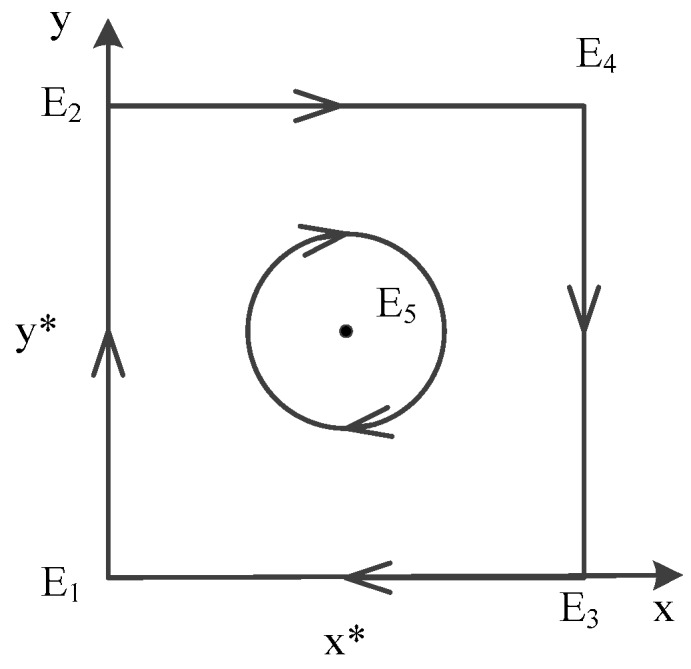
Diagram on dynamic evolution of equilibrium points in $E_{5}\left(x*,y*\right)$

**Figure 9:**
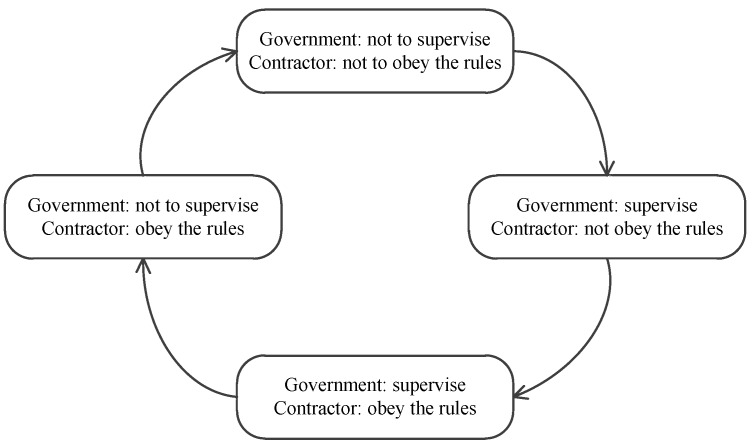
Circular process of evolutionary game between government and contractors in Case 12.

In Table 10, we find that $E_{1}\left(0,0\right)$, $E_{2}\left(0,1\right)$, $E_{3}\left(1,0\right)$, $E_{4}\left(1,1\right)$ are all unstable points, while $E_{5}\left(x*,y*\right)$ is a center point. The diagram on the dynamic evolution of equilibrium points (Figure 8) is shown that there is no ESS in this case. The ultimate choice changes dynamically and is affected by the initial strategy made by government and contractors. If the initial strategy between government and contractors is $E_{1}\left(0,0\right)$, the strategy will be changed to $E_{3}\left(1,0\right)$, then come to $E_{4}\left(1,1\right)$, and arrive at $E_{2}\left(0,1\right)$ with time going by. It is a circular process, and an ESS does not exist in this case. The circular process is depicted in Figure 9.

## 5. Discussion

Based on different stable points, there are 5 situations classified in 12 cases that are shown in Table 11. When the ESS is $E_{3}$, it means that the government does not need to supervise, and the contractors will obey the rules, which is the most ideal situation. When the ESS is $E_{4}$, it means the government supervises and the contractors obey the rules, which could be regarded as a suboptimal result that is also acceptable for the research.

### 5.1. Ultimate Strategy of {Obey The Rules, Not to Supervise}

Combining the prerequisites for case 7 and case 8, a wider prerequisite is concluded, which leads to $E_{3}$ as an ESS: $(\alpha_{2}-\alpha_{1})A-Ce>0$, $(k_{2}^{\prime }-k_{2})\alpha_{1}A-Cg<0$.

(1) $(\alpha_{2}-\alpha_{1})A-Ce>0$ is equivalent to $(\alpha_{2}-\alpha_{1})A>Ce$. $(\alpha_{2}-\alpha_{1})A$ is the difference of accident losses between obeying the rule and not obeying the rule by the contractors. The coefficients of $\alpha_{2}$, $\alpha_{1}$ and A are determined by the objective reality and they do not alter with the evolution. Ce represents the security cost of contractors which should be reduced.

(2) $\left(k_{2}^{\prime }-k_{2}\right)\alpha_{1}A-Cg<0$ is equivalent to $\left(k_{2}^{\prime }-k_{2}\right)\alpha_{1}A<Cg$. $\left(k_{2}^{\prime }-k_{2}\right)\alpha_{1}A$ is the difference of accident losses between supervising and not supervising by the government when the contractors obey the rules. Same as above, the coefficients of $k_{2}$, $k_{2}^{\prime }$, $\alpha_{1}$ and A are determined by the objective reality and they also do not alter with the evolution. Cg is the cost of safety supervision to the government. Then we should make sure that the value of Cg is greater than $\left(k_{2}^{\prime }-k_{2}\right)\alpha_{1}A$. At the same time, the value of Cg should be cut down by decreasing redundant costs to realize the best strategy.

### 5.2. Ultimate Strategy of {Obey The Rules, Supervise}

Combining the prerequisites for case 4, case 9, case 10 and case 11, a wider prerequisite is concluded, which leads to *E*_4_ as an ESS: $R-R^{\prime }+P_{1}+(\alpha_{2}-\alpha_{1})A-Ce>0$, $(k_{2}^{\prime }-k_{2})\alpha_{1}A-Cg>0$

(1) $R-R^{\prime }+P_{1}+(\alpha_{2}-\alpha_{1})A-Ce>0$ is equivalent to $(\alpha_{2}-\alpha_{1})A>R^{\prime }-R-P_{1}+Ce$. $(\alpha_{2}-\alpha_{1})A$ is the difference of accident losses between obeying the rule and not obeying the rule by the contractors. The coefficients of $\alpha_{1}$, $\alpha_{2}$ and A are determined by the objective reality. They have no relationship with the state of evolution. The only way to make this inequality work is to minimize the value of the term at the right side. $R^{\prime }-R$ is the difference of contractors’ business revenue between supervising and not supervising by the government. $P_{1}$ represents administrative penalties under the condition that contractors do not obey the rules and government supervises. Ce is the security cost of the contractors. The value of $R^{\prime }$ and Ce should be increased, the value of R and $P_{1}$ should be decreased to minimize the value of the right term.

(2) $\left(k_{2}^{\prime }-k_{2}\right)\alpha_{1}A-Cg>0$ is equivalent to $\left(k_{2}^{\prime }-k_{2}\right)\alpha_{1}A>Cg$. $\left(k_{2}^{\prime }-k_{2}\right)\alpha_{1}A$ is the difference of accident losses whether the government supervises or not when the contractors obey the rules. Same as above, the coefficients of $k_{2}$,$k_{2}^{\prime }$,$\alpha_{1}$ and A are determined by the objective reality and do not alter with the evolution. Cg is the cost of safety supervision to the government. Contrary to the ultimate strategy of {obey the rules, not to supervise}, we do not need to consider the value of $\left(k_{2}^{\prime }-k_{2}\right)\alpha_{1}A$, we just need to minimize the value of Cg.

### 5.3. Parameters in This Evolutionary Game Model

Through the above discussion about two ultimate strategies we want, the parameters involved are Ce, Cg, R, R′, $P_{1}$. Among them, the value of Ce, Cg, R′ should be decreased, while the value of R, $P_{1}$ should be increased.

R is the normal business revenue of contractors and its increase is beyond the scope of our discussion. $P_{1}$ presents administrative penalty with little success. The reform of the economic system in China should focus on the market-oriented allocation of resources. According to this reform thought, administrative penalties may be reduced and the value of $P_{1}$ will remain the same or lower. The safety information system in the construction industry would replace the partial effect of administrative penalties, and the parametric functions of it are as follows:

(1) Decrease the value of Ce. To contractors who obey the rules and with a top safety performance ranking according to the safety information system, the government and some nongovernmental organizations may use part of their budget as bonuses. Enforcing market-oriented policy tools (financial incentives like a subsidy or even tax deductions) for contractors with good safety performance to reduce their safety construction costs can be a great help in promoting healthy competition in the construction market.
(2) Decrease the value of R′. By applying a safety information system in the construction industry, it is more convenient for the public to participate in the supervision. Therefore, the expected revenues of contractors would decrease significantly once they do not obey the rules and have bad performance records. As the economic income of contractors would be greatly affected by their credit rating, then the probability of rule-breaking behaviors would also be reduced, creating a positive safety climate for the construction industry.
(3) Decrease the value of Cg. Through the above analysis, it is essential to promote the public participating in the supervision and to reduce the pressure on government safety supervision with the help of market forces, thereby improving the overall efficiency of social regulatory. That is why the value of Cg could decrease dramatically.

## 6. Conclusions

Taking Construction Safety Performance in China as the context, unlike previous research that elaborated safety climate factors and the advantages of the safety regulations [2,32,45], this paper explores the internal mechanism of market forces to further strengthen the management level of safety supervision in China. In order to improve safety performance and reduce occupational injuries in construction industry, we proposed a conception of a safety information system (the blacklist) by several brainstorming sessions from a group of professionals. Therefore, we proposed this as a new path to improve construction safety performance in China. Relevant government departments and a wide range of nongovernmental organizations are the basic sources of credit information. The safety information system will provide rating information to the public, as bad performance contractors will be added to the blacklist, which can seriously influence their economic activities. The purpose of establishing the blacklist for safe production of construction enterprises is not entirely to implement disciplinary action, but also to conduct market-oriented supervision, which aims to have more benign competition in the construction industry market to spontaneously survive the inferior construction companies, and to allow contractors realize that safety performance is extremely important for their survival. Therefore, the aim is to stimulate these companies to be responsible for improving their safety performance.

In order to examine the function of the blacklist, we proposed an evolutionary game-theoretic model that aims to investigate when government and contractors choose optimal behavioral strategies. We determined 12 stable states of this model and 5 possible evolutionary stable equilibrium points in this method. Then, the local stability of equilibrium was carefully deduced in each case, and the dynamic evolution diagram was drawn through calculation results. In particular, we focused on two evolutionary stable equilibrium points, which are that contractors and government choose {obey the rules, not to supervise} and {obey the rules, supervise} respectively.

Based on these situations, some novel results have been obtained by theoretical analysis: the main factors influencing the behavioral strategies of government and contractors are Ce (cost of safety construction to the contractors who obey the rules), Cg (cost of safety supervision to the government) and R′ (revenue to the contractors when they do not obey the rules and the government have the safety supervision), and the value of them should be reduced to ensure the evolution strategies for the ESSs. The discussion section above leads to the best situations of this evolutionary model, which verifies the success of the safety information system. This paper found that the safety information system can help in reducing the value of Ce, Cg and R′ through the internal mechanism of market forces.

This safety information system in construction has significant value as the Chinese construction supervision is undergoing a transformation from a “nanny-style” administrative mode to a market-oriented governance mode. Corresponding to the defects of “nanny-style” administrative mode, the benefits of market-oriented governance mode are illustrated as follows:

- On the one hand, the market-oriented governance mode encourages the public to participate in the supervision system, thereby reducing the safety supervision pressure on the government. Market forces help to reduce the labor and material resources that the government needs to invest in safety supervision to improve the overall efficiency of social regulatory. On the other hand, the efficiency of safety regulation is higher not only thanks to government forces, but also taking market forces as a supplementary supervision method, realizing the optimal allocation of social resources.
- Although the antagonistic relationship between the supervisors and the regulated parties still exists, with the help of market forces, even under various situations, they can eventually reach ESS points. This can effectively boost the enthusiasm and consciousness of participants’ responsibility, which is conducive to the sustainable and healthy development of safety supervision.

Based on the conclusions above, this paper also proposes several practical suggestions for government policy makers to improve construction safety performance and to reduce occupational injuries in China. (1) The government should gladly adapt to various market conditions. In order to ensure effective construction safety management, government should improve the management level, supervisory strength and reducing the supervision costs at the same time. It is essential for local construction administrative departments to take specific measures according to the development levels of certain areas. (2) The government should reduce the safety construction costs of contractors and continuously strengthen the incentives like tax deductions for contractors to adopt benign safety behaviors in the pursuit of long-term interests. (3) The government should take note of the revealing of construction safety information to society. The government should pay particularly close attention to the accuracy and timeliness of safety information sources. The government should also make full use of the public supervision embedded with its justice, honesty and credibility.

However, there are still some limitations in our study. Research can be further studied from multiple perspectives. First, our evolutionary game model takes contractors into consideration because they are the main management objects to supervise by the government, but in reality, better safety performance in the construction industry needs joint efforts from all the stakeholders, including government, contractors, designers, supervisors and so on. Therefore, there are more complex factors that would influence the optimal behavioral strategies of participants. Second, the effect of public participation in safety supervision has not been quantified in this paper, so further research can pay attention to analyze the effect of market-oriented supervision through quantitative methods.

## Figures and Tables

**Table 1:** Summary of notations.

Symbol	Description
R	Normal business revenue to the contractors, R>0
$R^{\prime }$	Revenue to the contractors when they do not obey the rules and the government have the safety supervision, $R>R^{\prime }>0$
Ce	Cost of safety construction to the contractors who obey the rules, Ce>0
Cg	Cost of safety supervision to the government, Cg>0
A	Biggest loss caused by safety accidents, A>0
$\alpha_{1}$	Accident probability when contractors obey the rules, $\alpha_{1}>0$
$\alpha_{2}$	Accident probability when contractors do not obey the rules, $\alpha_{2}>\alpha_{1}>0$
$P_{1}$	All types of administrative penalties which government imposes by adopting supervisory measures when contractors do not obey the rules, $P_{1}>0$
$P_{2}$	The government revenues, $P_{1}>P_{2}>0$
$k_{1}$	Benefit coefficient of government which based on the assumption that the government’s benefits are positively correlated with the normal business revenue to the contractors, $k_{1}>0$
$k_{2}$	Safety accident cost coefficient to the government when the government supervises contractors, $k_{2}>0$
$k_{2}^{\prime }$	Safety accident cost coefficient to the government when the government does not supervise contractors, $k_{2}^{\prime }>k_{2}>0$

**Table 2:** Payoff matrix between government and contractors.

Contractors	Government	
	Supervise	Not Supervise
Obey	$R-Ce-\alpha_{1}A,\;k_{1}R-Cg-k_{2}\alpha_{1}A$	$R-Ce-\alpha_{1}A,\;k_{1}R-k_{2}^{\prime }\alpha_{1}A$
Not Obey	$R\prime -\alpha_{2}A-P_{1},\;k_{1}R^{\prime }-Cg-k_{2}\alpha_{2}A+p_{2}$	$R-\alpha_{2}A,\;k_{1}R-k_{2}^{\prime }\alpha_{2}A$

**Table 3:** Judging standard of ESS.

Sign of det*J*	Sign of tr*J*	Result
Negative	Uncertain	A saddle points.
Positive	Positive	An unstable point.
Positive	Negative	A stable point. An ESS is found.

**Table 4:** Equation of det*J* and tr*J* of five possible equilibrium points.

Equilibrium	Equation of det*J* and tr*J*
E**_1_**(0,0)	$\det J=[(\alpha_{2}-\alpha_{1})A-Ce][(k_{2}^{’}-k_{2})\alpha_{2}A-k_{1}(R-R^{’})-Cg+P_{2}]$
	$trJ=[(\alpha_{2}-\alpha_{1})A-Ce]+[(k_{2}^{’}-k_{2})\alpha_{2}A-k_{1}(R-R^{’})-Cg+P_{2}]$
E**_2_**(0,1)	$\det J=-[R-R^{’}+P_{1}+(\alpha_{2}-\alpha_{1})A-Ce][(k_{2}^{’}-k_{2})\alpha_{2}A-k_{1}(R-R^{’})-Cg+P_{2}]$
	$trJ=[R-R^{’}+P_{1}+(\alpha_{2}-\alpha_{1})A-Ce]-[(k_{2}^{’}-k_{2})\alpha_{2}A-k_{1}(R-R^{’})-Cg+P_{2}]$
E**_3_**(1,0)	$\det J=-[(\alpha_{2}-\alpha_{1})A-Ce][(k_{2}^{’}-k_{2})\alpha_{1}A-Cg]$
	$trJ=-[(\alpha_{2}-\alpha_{1})A-Ce]+[(k_{2}^{’}-k_{2})\alpha_{1}A-Cg]$
E**_4_**(1,1)	$\det J=[R-R^{’}+P_{1}+(\alpha_{2}-\alpha_{1})A-Ce][(k_{2}^{’}-k_{2})\alpha_{1}A-Cg]$
	$trJ=-[R-R^{’}+P_{1}+(\alpha_{2}-\alpha_{1})A-Ce]-[(k_{2}^{’}-k_{2})\alpha_{1}A-Cg]$
E**_5_**(x*,y*)	$\det J=\frac{\left[(k_{2}^{’}-k_{2})\alpha_{2}A-k_{1}(R-R^{’})-Cg+P_{2}][(k_{2}^{’}-k_{2})\alpha_{1}A-Cg][(\alpha_{2}-\alpha_{1})A-Ce][R-R^{’}+P_{1}+(\alpha_{2}-\alpha_{1})A-Ce\right]}{\left[(k_{2}^{’}-k_{2})(\alpha_{2}-\alpha_{1})A-k_{1}(R-R^{’})+P_{2}][R-R^{’}+P_{1}\right]}$
	trJ=0

**Table 5:** Representation of formulas.

Symbol	Formula	Symbol	Formula
a	$(\alpha_{2}-\alpha_{1})A-Ce$	c	$(k_{2}^{’}-k_{2})\alpha_{2}A-k_{1}(R-R^{’})-Cg+P_{2}$
b	$R-R^{’}+P_{1}+(\alpha_{2}-\alpha_{1})A-Ce$	d	$(k_{2}^{’}-k_{2})\alpha_{1}A-Cg$

**Table 6:** Proposition 1: Analysis of local stability of equilibrium.

Equilibrium	Case 1			Case 2-1		
	det*J*	tr*J*	Result	det*J*	tr*J*	Result
E1(0,0)	+	-	Stable	+	-	Stable
E2(0,1)	-	±	Saddle	+	+	Unstable
E3(1,0)	-	±	Saddle	-	±	Saddle
E4(1,1)	+	+	Unstable	-	±	Saddle
		**Case 2-2**			**Case 3**	
E1(0,0)	+	-	Stable	+	-	Stable
E2(0,1)	+	+	Unstable	-	±	Saddle
E3(1,0)	-	±	Saddle	+	+	Unstable
E4(1,1)	-	±	Saddle	-	±	Saddle
E5(x*,y*)	+	0	Center			
		**Case 4**				
E1(0,0)	+	-	Stable			
E2(0,1)	+	+	Unstable			
E3(1,0)	+	+	Unstable			
E4(1,1)	+	-	Stable			

Note: + represents the sign is positive; - represents sign is negative; ± represents the sign is uncertain.

**Table 7:** Proposition 2: Analysis of local stability of equilibrium.

Equilibrium	Case 5			Case 6		
	det*J*	tr*J*	Result	det*J*	tr*J*	Result
E1(0,0)	-	±	Saddle	-	±	Saddle
E2(0,1)	+	-	Stable	+	-	Stable
E3(1,0)	-	±	Saddle	+	+	Unstable
E4(1,1)	+	+	Unstable	-	±	Saddle

Note: + represents the sign is positive; - represents sign is negative; ± represents the sign is uncertain.

**Table 8:** Proposition 3: Analysis of local stability of equilibrium.

Equilibrium	Case 7			Case 8		
	det*J*	tr*J*	Result	det*J*	tr*J*	Result
E1(0,0)	-	±	Saddle	+	+	Unstable
E2(0,1)	+	+	Unstable	-	±	Saddle
E3(1,0)	+	-	Stable	+	-	Stable
E4(1,1)	-	±	Saddle	-	±	Saddle

Note: + represents the sign is positive; - represents sign is negative; ± represents the sign is uncertain.

**Table 9:** Proposition 4: Analysis of local stability of equilibrium.

Equilibrium	Case 4			Case 9		
	det*J*	tr*J*	Result	det*J*	tr*J*	Result
E1(0,0)	+	-	Stable	-	±	Saddle
E2(0,1)	+	+	Unstable	+	+	Unstable
E3(1,0)	+	+	Unstable	-	±	Saddle
E4(1,1)	+	-	Stable	+	-	Stable
		**Case 10**			**Case 11**	
E1(0,0)	-	±	Saddle	+	-	Unstable
E2(0,1)	-	±	Saddle	-	±	Saddle
E3(1,0)	+	+	Unstable	-	±	Saddle
E4(1,1)	+	-	Stable	+	-	Stable

Note: + represents the sign is positive; - represents sign is negative; ± represents the sign is uncertain.

**Table 10:** Proposition 5: Analysis of local stability of equilibrium.

Equilibrium	Case 12		
	det*J*	tr*J*	Result
E1(0,0)	-	±	Unstable
E2(0,1)	-	±	Unstable
E3(1,0)	-	±	Unstable
E4(1,1)	-	±	Unstable
E5(x*,y*)	+	0	Center

Note: + represents the sign is positive; - represents sign is negative; ± represents the sign is uncertain.

**Table 11:** Category of cases based on stable points.

Stable Point	Category	Ultimate Strategy
$E_{1}$	Case 1, Case 2, Case 3, Case 4	{not to obey the rules, not to supervise}
$E_{2}$	Case 5, Case 6	{not to obey the rules, supervise}
$E_{3}$	Case 7, Case 8	{obey the rules, not to supervise}
$E_{4}$	Case 4, Case9, Case10, Case 11	{obey the rules, supervise}
None	Case 12	Circular process
